# Immune molecule diagnostics in colorectal cancer: CCL2 and CXCL11

**DOI:** 10.1515/biol-2025-1147

**Published:** 2025-08-08

**Authors:** Hui Zhang, Baohong Xu, Mengqi Yin, Yan Dong, Mingliang Lu

**Affiliations:** Department of Gastroenterology, Wu Han No. 1 Hospital, WuHan, HuBei, China; Department of Gastroenterology, Beijing Luhe Hospital, Capital Medical University, Beijing, 101149, China; Department of Neurology, Wu Han No. 1 Hospital, WuHan, HuBei, China; Pathology Department, The Third Affiliated Hospital of Kunming Medical University and Yunnan Cancer Center, Kunming, Yunnan, China; Department of Gastroenterology, Beijing Luhe Hospital, Capital Medical University, Beijing, 101149, China

**Keywords:** sessile serrated adenoma, traditional serrated adenoma, colorectal cancer, CCL2, CXCL11, molecular diagnosis

## Abstract

Traditional serrated adenomas (TSAs) and sessile serrated adenomas (SSAs) are known precursors to colorectal cancer (CRC), but differentiating between them morphologically can be challenging. This study developed an immune molecule-based model to distinguish TSA from SSA using RNA sequencing data from the GEO datasets (GSE117606, GSE45270, GSE117607). Gene expression profiling was conducted with the R package GEOquery, and immune cell infiltration was assessed using CIBERSORTx. Differential expression analysis of immune-related genes was performed with the “limma” package. Enrichment analysis of differentially expressed genes (DEGs) was conducted using “clusterProfiler” for Gene Ontology and kyoto encyclopedia of genes and genomes pathways, identifying protein-protein interaction networks to find core hub genes. Notable differences in immune cell infiltration were observed among SSA, TSA, CRC, and healthy tissues, involving various immune cell types. A total of 45 DEGs (34 upregulated, 11 downregulated) were identified, with CCL2 and CXCL11 emerging as key hub genes. Their diagnostic potential was validated through receiver operating characteristic analysis in GEO datasets and clinical samples, while immunohistochemistry revealed decreased expression of CCL2 and CXCL11 in SSA compared to TSA and normal tissues, indicating their role in SSA pathogenesis and potential as molecular diagnostic markers. The diagnostic value of CCL2 is superior to that of CXCL11, while the diagnostic value of CXCL11 requires further experimental verification.

## 1 Introduction

Colorectal cancer (CRC), also known as colon cancer, bowel cancer, or rectal cancer, is the most common type of gastrointestinal cancer [1]. It is crucial to identify lesions before the tissue transforms because most CRC cases are caused by the malignant transformation of precancerous adenomatous and serrated polyps (2).

Serrated polyps are usually classified into four categories, i.e., hyperplastic polyps, sessile serrated adenomas (SSAs), traditional serrated adenomas (TSAs), and unclassified serrated adenomas, among which SSA with dysplasia and TSA are the most common precursors of CRC [2]. The serrated polyps that are most important are those that are slightly flattened, broadly based, and contain small, round to oval, and basally located nuclei [3]. SSA is a complicated disease that interacts with the microenvironment, usually located on the crests of mucosal folds [3]. The molecular landscape of sessile serrated lesions is characterized by wild-type *KRAS* gene, mutated v-raf murine sarcoma viral oncogene homolog B gene (or *BRAF*), CpG-island methylator-H phenotype (or CIMP-H phenotype) with methylated Human *Mut-L* Homologue 1 (also known as *MLH1*), which are acquired in an early phase and result in microsatellite instability (MSI) or methylated hypermethylation of O^6^-methylguanine DNA methyltransferase (also known as *MGMT*) with WNT pathway activation and MSS status. TP53 mutation and p16 silencing are found in only a small number of instances [4]. TSA, also known as a “pinecone-like” lesion, is a rare condition that occurs in less than 1% of all serrated polyps and less than 1% of all colorectal polyps [5]. The molecular heterogeneity of TSA lesions is present, with KRAS mutations being slightly less prevalent than BRAF mutations. TSA is also characterized by a CIMP-L phenotype. When they develop high-grade dysplasia, they may present activation of the WNT pathway and mutations in TP53 [6]. Moreover, some studies [7,8] have suggested that SSA is a recognized precursor of CRC with high MSI levels, whereas TSA is more likely to evolve into a TSA that is microsatellite-stable or has low levels of MSI. Even though SSA and TSA are distinct (demographically and molecularly) [9], it can be challenging to distinguish them by only cytologic characteristics.

In this study, we established and validated a novel molecular diagnostic model based on the immune response, which was used to differentiate SSA from TSA.

## 2 Materials and methods

### 2.1 Microarray datasets and data processing

SSA and TSA data were collected from the GSE117606 database (gene expression profiles by array from 70 patients), which consists of 10 SSAs, 59 TSAs, 74 tumor tissues, and 65 adjacent normal tissues; the GSE45270 database (gene expression profiles by array from patients, including 6 serrated adenomas and 7 tubular adenomas); and GSE117607 database (gene expression profiles by array from 135 patients presenting with colorectal adenomas during surgery or colonoscopy). All the GEO datasets were downloaded and processed using the R package GEOquery.

### 2.2 Immune infiltration analysis

The evaluation of 22 immune cell infiltration levels was done using CIBERSORT and R language. As previously reported [10], the proportion of immune cells in each sample was calculated.

### 2.3 Identification of immune-related differentially expressed genes (DEGs)

The DEGs between tissues (SSAs vs TAs and SSAs vs normal intestinal tissues) were analyzed using the “limma package” in R language. Genes with a *P*-value of <0.05 were considered significant; immune-related DEGs were considered overlaps with immune cell-specific marker genes, as selected in the R language [11].

### 2.4 Functional and pathway enrichment analysis

The functions of DEGs were analyzed using Gene Ontology (GO) analysis. The “clusterProfiler” package was used to conduct GO analysis [12]; a *P*-value of <0.05 was considered statistically significant enrichment. Kyoto encyclopedia of genes and genomes (KEGG) analysis was used to assess significant pathways for gene enrichment.

### 2.5 Protein–protein interaction (PPI) network analysis of immune-related DEGs

PPIs among immune-related DEG proteins were examined using the STRING platform [13]. Sub-clusters of the PPI network were assessed using the Cytoscape plug-in. Consequently, genes with high scores were selected. The intersection of the results from ten different algorithms was evaluated, and the top two highest-scoring genes were extracted for further analysis.

### 2.6 Inclusion criteria


**Inclusion criteria:** Patients diagnosed with serrated adenoma or traditional adenoma.
**Specimen processing and analysis:** Cutting slides from formalin-fixed, paraffin-embedded blocks, followed by immunohistochemical analysis. The entire procedure is non-invasive to the patient.

### 2.7 Immunohistochemistry (IHC) assay

The clinical samples obtained from patients with SSAs (*n* = 6), patients with TSAs (*n* = 6), and patients with normal intestinal tissues (*n* = 6) were collected from Yunnan Cancer Center (Kunming, China) from 2019 to 2021. After pretreating the samples, they were incubated with primary antibodies against MCP1 Polyclonal antibody (1:200 dilution; Rabbit; no. 25542-1-AP; Proteintech) and CXCL11 Polyclonal antibody (1:200 dilution; Rabbit; no. 10707-1-AP; Proteintech). Image-Pro Plus 6.0 (Media Cybernetics, Inc., Rockville, MD, USA) was used for analysis, after which the mean optical density (MOD) value was calculated.


**Informed consent:** Informed consent has been obtained from all individuals included in this study.
**Ethical approval:** The research related to human use has been complied with all the relevant national regulations and institutional policies and in accordance with the tenets of the Helsinki Declaration and has been approved by the Ethics Committee of The Third Affiliated Hospital of Kunming Medical University and Yunnan Cancer Center.

### 2.8 Statistical analysis

The analysis of variance (ANOVA) method was applied to compare the continuous variables among the three groups. The Student’s *t*-test was used to compare the continuous variables of the two groups in clinical features. A non-parametric test was applied if the data presented dissatisfied homogeneity of variance. Pearson’s analysis was used to analyze the correlation between gene expression and the fraction of immune cells. The diagnostic value of gene expression in patients with SSAs was analyzed using receiver operating characteristic (ROC) curves, with the area under curve (AUC) being used to estimate the diagnostic value. The statistical analyses were carried out using R software (version 4.1.0). Image analysis: Grayscale quantification of stained tissue sections was performed using ImageJ software (v1.53). Data acquisition: a minimum of five sample points were systematically collected within identical regions of interest. Calculation: MOD values were computed using the formula:

\[\text{MOD}=\frac{\sum (\text{optical}\hspace{.25em}\text{density}\hspace{.25em}\text{values})}{n},]\]

where *n* represents the number of measurement points. Statistical comparison: Intergroup comparisons were conducted using one-way ANOVA with post hoc Tukey’s test. *P*-value <0.05 was considered statistically significant.

## 3 Results

### 3.1 Landscape of immune cell infiltration in SSA and TSA

**Figure 1:**
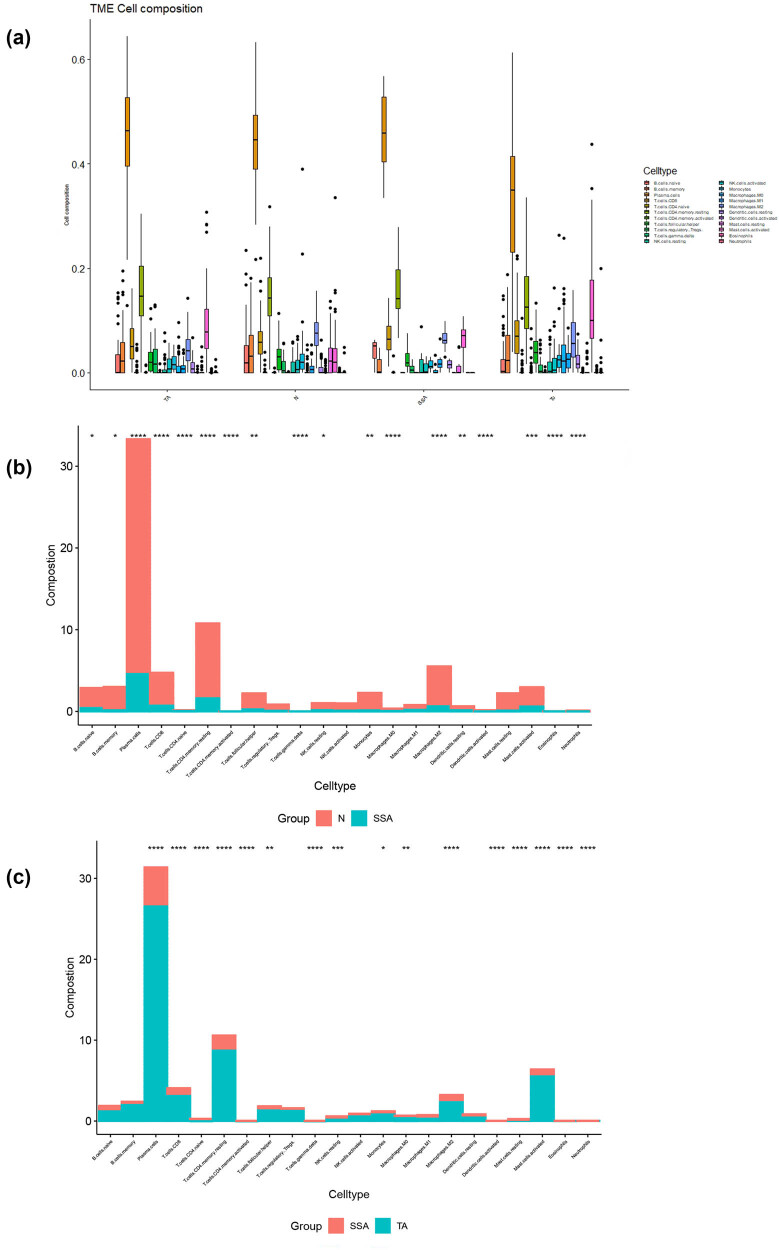
(a) Landscape of 22 immune cell subgroups infiltration in four CRC tissues. Proportion of immune cell infiltration between SSA and N (b) and between SSA and TSA (c). SSA: sessile serrated adenoma; TSA: traditional serrated adenoma; N: normal tissue.

We first assessed the landscape of cell infiltration in SSA, TA, tumors, and adjacent normal tissues using CIBERSORTx.22. The sub-populations of immune cells were obtained from the GSE117606 dataset, which contained 10 SSAs, 59 TSAs, 74 tumor tissues, and 65 adjacent normal tissues (N tissues). As expected, obvious differences were seen in the four groups (Figure 1a): activated and resting mast cells, activated and resting dendritic cells, regulatory T cells (Tregs), macrophages (M1, M2, and M3), follicular helper T cells, activated memory T CD4 cells, plasma cells, and naive B cells showed statistically significant differences by *K*–*W* analysis (*P* < 0.05; Table 1). The proportion of cell infiltration between SSA and TA and between SSA and *N* was clearly different (Figure 1b and c).

**Table 1:** Immune cell infiltration showed statistically significant differences among the four groups

Immune cells	*P* values
Activated mast cells	1.53 × 10^−13^
Resting mast cells	1.82 × 10^−16^
Activated dendritic cells	0.01
Resting dendritic cells	1.73 × 10^−7^
**Macrophages**	
M0	1.72 × 10^−8^
M1	4.43 × 10^−9^
M2	1.28 × 10^−5^
Regulatory T cells (Tregs)	0.02
Follicular Helper T cells	0.003
Activated memory T CD4 cells	0.0002
Plasma cells	7.92 × 10^−11^
Naive B cells	0.01

### 3.2 DEG and GO/KEGG analyses of immune-related genes

**Figure 2:**
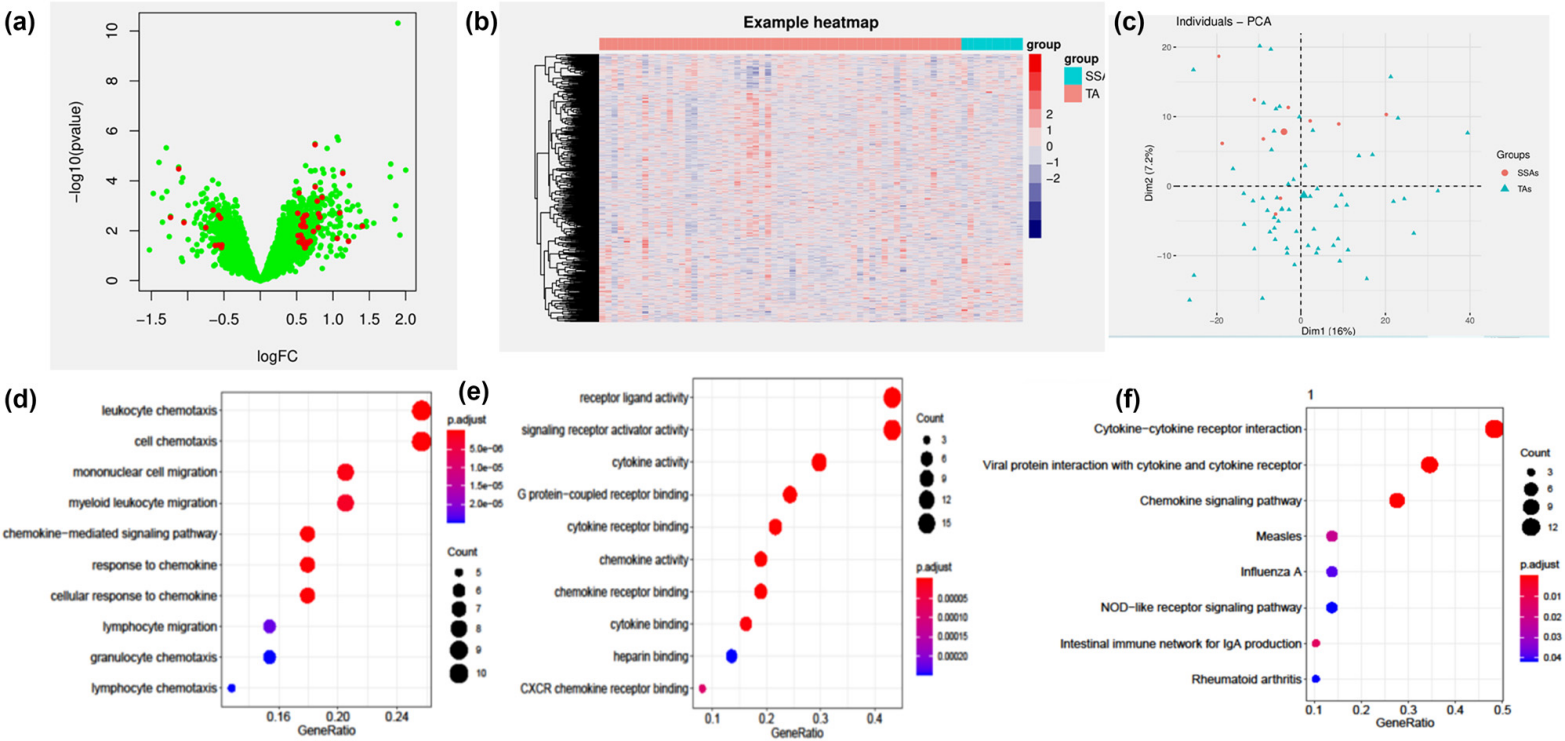
(a) Volcano plot of immune-related genes in GSE117606 (red represents DEGs). (b) The heatmap shows immune-related genes in SSA and TA. (c) Principal component analysis (PCA) involving immune-related genes in GSE117606. (d and e) Biological process (BP) and molecular function (MF) of immune-related DEGs between TA and SSA, respectively. (f) KEGG analysis of immune-related DEGs between TA and SSL.

After screening overlaps (first, immune-related genes were screened; then, the differences between pathological and healthy tissues were analyzed), 1,092 genes were associated with the immune system. Then, DEG analysis (*P*-value <0.05 and logFCcutoff = 0.5) between SSA and TSA in the GSE117606 dataset was conducted. DEGs were defined (*P* < 0.05 and logFCcutoff = 0.5), and 45 DEGs (34 up- and 11 downregulated genes) were obtained (Figure 2a). A heatmap shows immune-related genes in SSA and TA (Figure 2b). Figure 2c shows the PCA analysis between the two groups.

The results of GO/KEGG analysis revealed that the genes were mainly involved in signaling receptor activator activity (GO: 0030546), the molecular function receptor ligand activity (GO: 0048018), cytokine activity (GO: 0005125), cell chemotaxis (GO: 0060326), and biological process leukocyte chemotaxis (GO: 0030595) (Figure 2d and e). No statistical value was noted for CC. KEGG analysis displayed that genes are involved in the cytokine-cytokine receptor interaction, chemokine signaling pathway, and viral protein interaction with cytokine and cytokine receptors (Figure 2f).

### 3.3 PPI network for key immune-related gene selection

**Figure 3:**
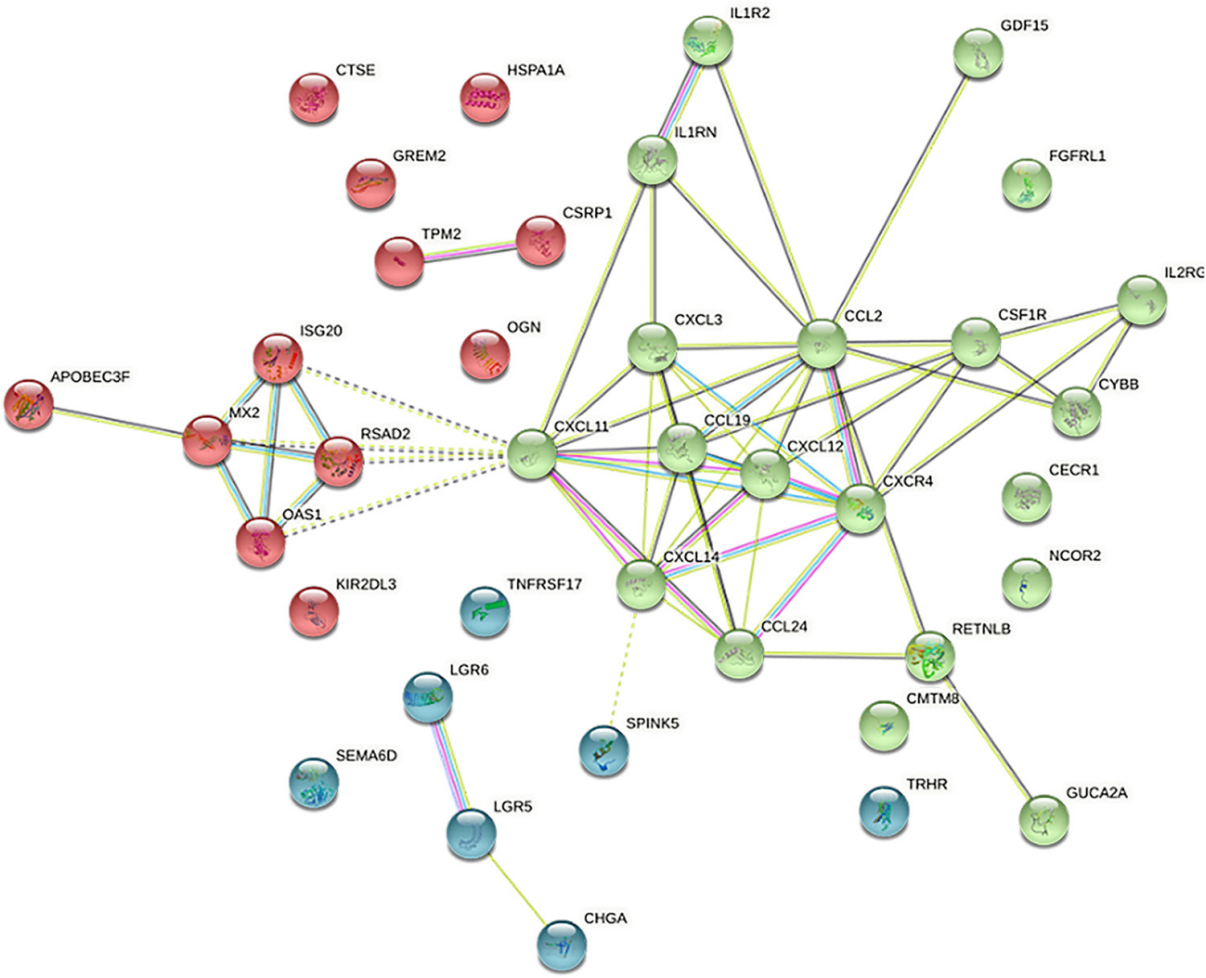
PPI network for DEGs.

A PPI network with 45 DEGs was created to confirm the protein interaction between the immune-related genes (Figure 3). Two hub genes, CCL2 and CXCL11, were identified by the intersection of 10 algorithms using CytoHubba, with the highest score, and sub-cluster scores of 7.714 (Table 2, Figure 3).

**Table 2:** Ten different algorithms for hub genes that had the highest scores by CytoHubba

Algorithm	Gene1	Gene2	Gene3	Gene4	Gene5
MCC	CXCL14	CCL24	CXCL12	CCL19	CXCL11
DMNC	CCL2	CXCR4	CXCL12	CCL19	CXCL11
MNC	CCL2	CXCL11	CXCR4	CXCL12	CCL19
Degree	CXCL11	CCL2	CXCR4	CXCL3	CCL19
EPC	CXCL11	CCL2	CXCR4	CCL24	LGR5
BottleNeck	CCL11	CCL2	CXCR4	CCL24	CXCL14
EcCentricity	CXCL11	CCL2	CXCR4	CXCL14	CXCL12
Closeness	CXCL11	CCL2	CXCR4	CXCL14	CXCL12
Radiality	CXCL11	CCL2	CXCL14	RETNLB	MX2
Betweenness	CCL2	CXCL11	RETNLB	CXCR4	CXCL14

### 3.4 Correlation between selected genes and immune cells in SSA

**Figure 4:**
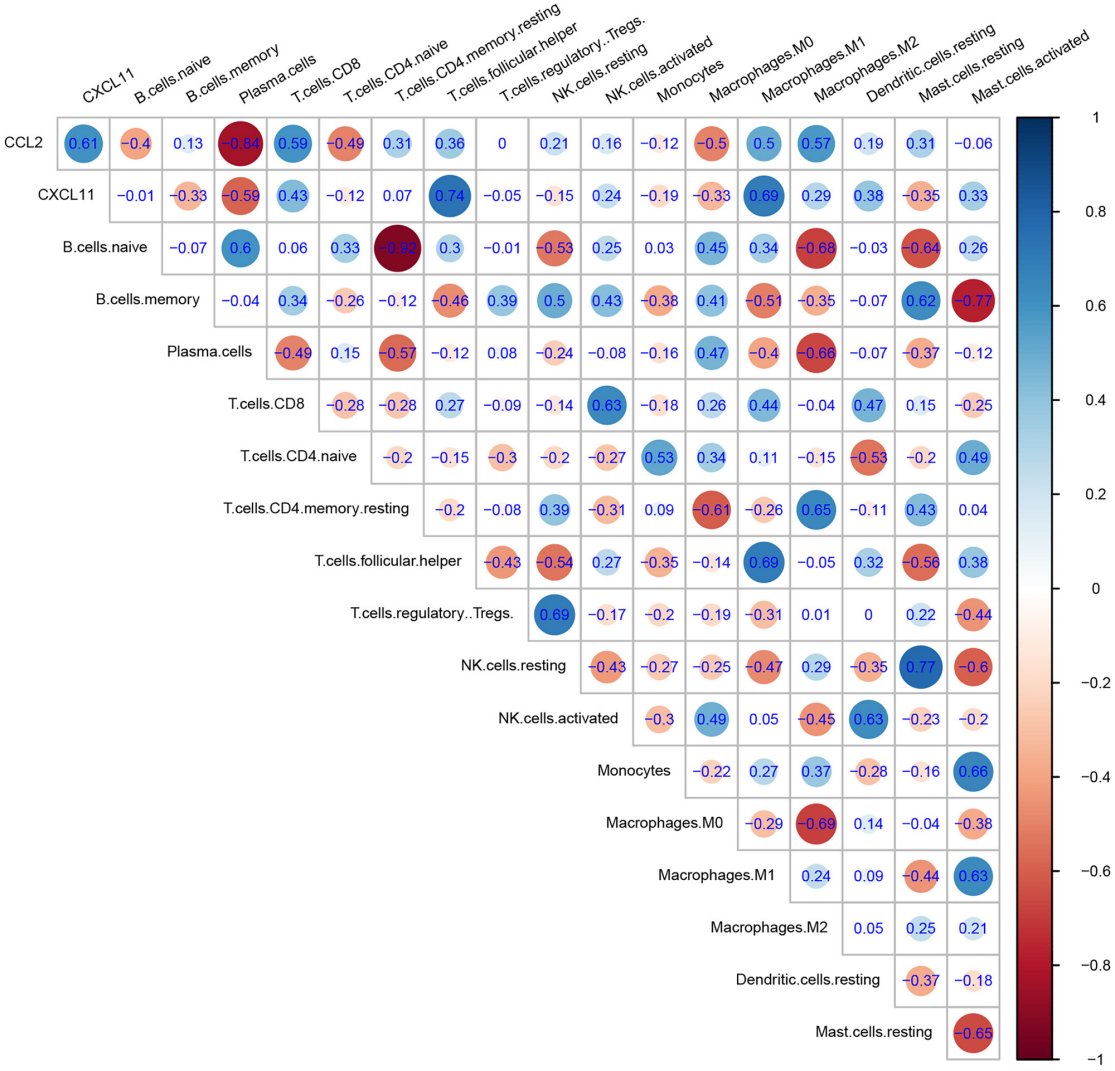
Correlation analysis among hub genes and immune-related cells in SSA.

We conducted a correlation analysis between selected hub genes and 22 immune cells in SSA, as shown in Figure 4. *CCL2* showed a strong correlation with plasma cells and a moderate correlation with M0, M1, and M2 macrophages, as well as CD8 T and CD4 naive T cells. CXCL11 showed a strong correlation with follicular helper T cells and M1 macrophages and a moderate correlation with CD8 T cells and plasma cells (Figure 4). Both hub genes are related to plasma cells, CD8 T cells, and M1 macrophages.

### 3.5 Diagnostic value of *CCL2* and *CXCL11*

The diagnostic value of CCL2 and CXCL11 was estimated using GSE117606, a training dataset.

**Figure 5:**
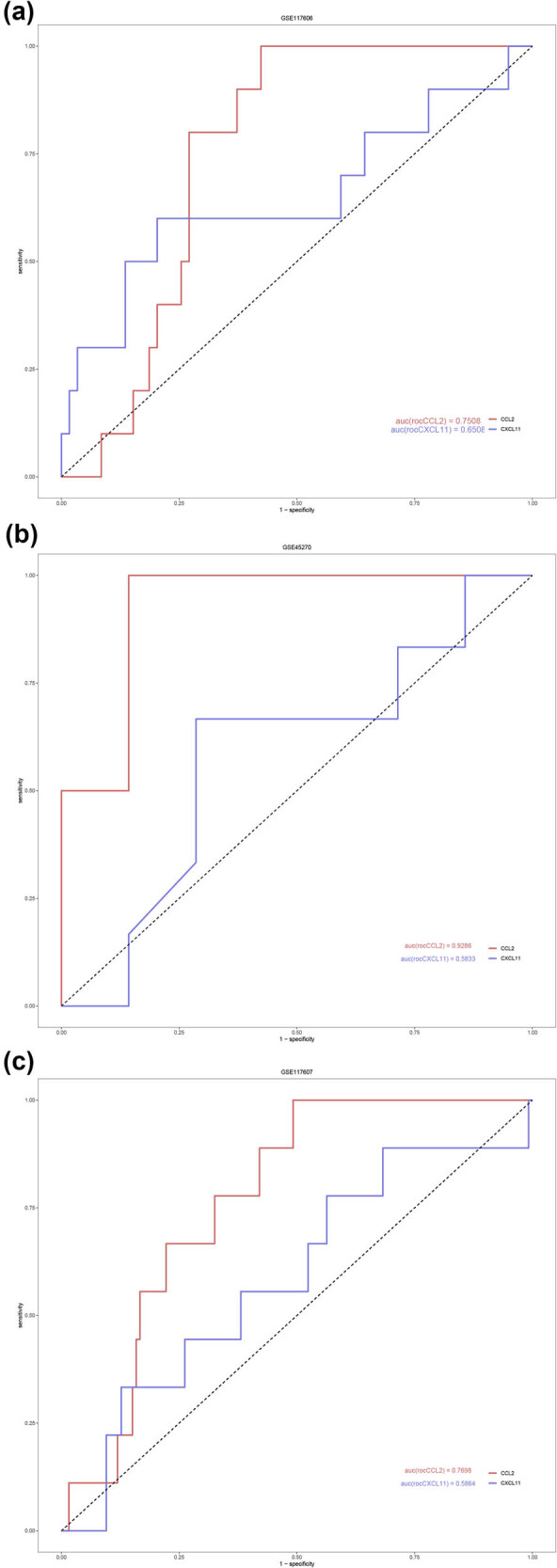
ROC curves of CCL2 (red line) and CXCL11 (blue line) in different GSE datasets: (a) GSE117606, (b) GSE117607, and (c) GSE45270 datasets.

CCL2 and CXCL11 in SSA had AUC values of 0.7508 and 0.6508, respectively (Figure 5a), which suggests that these genes have a greater potential for diagnosing SSA. Our conclusion was verified using the GSE45270 and GSE117607 datasets. The diagnostic values were similar to GSE117606; the AUC values of *CCL2* in the datasets GSE45270 (Figure 5b) and GSE117607 datasets (Figure 5c) were 0.9286 and 0.7698, respectively, while the AUC values of *CXCL11* in the GSE45270 and GSE117607 datasets were 0.5833 and 0.5864, respectively. The *CCL2* showed a better effect than *CXCL11* in distinguishing SSA from TA.

### 3.6 Expression of CCL2 and CXCL11 in SSA tissues

**Figure 6:**
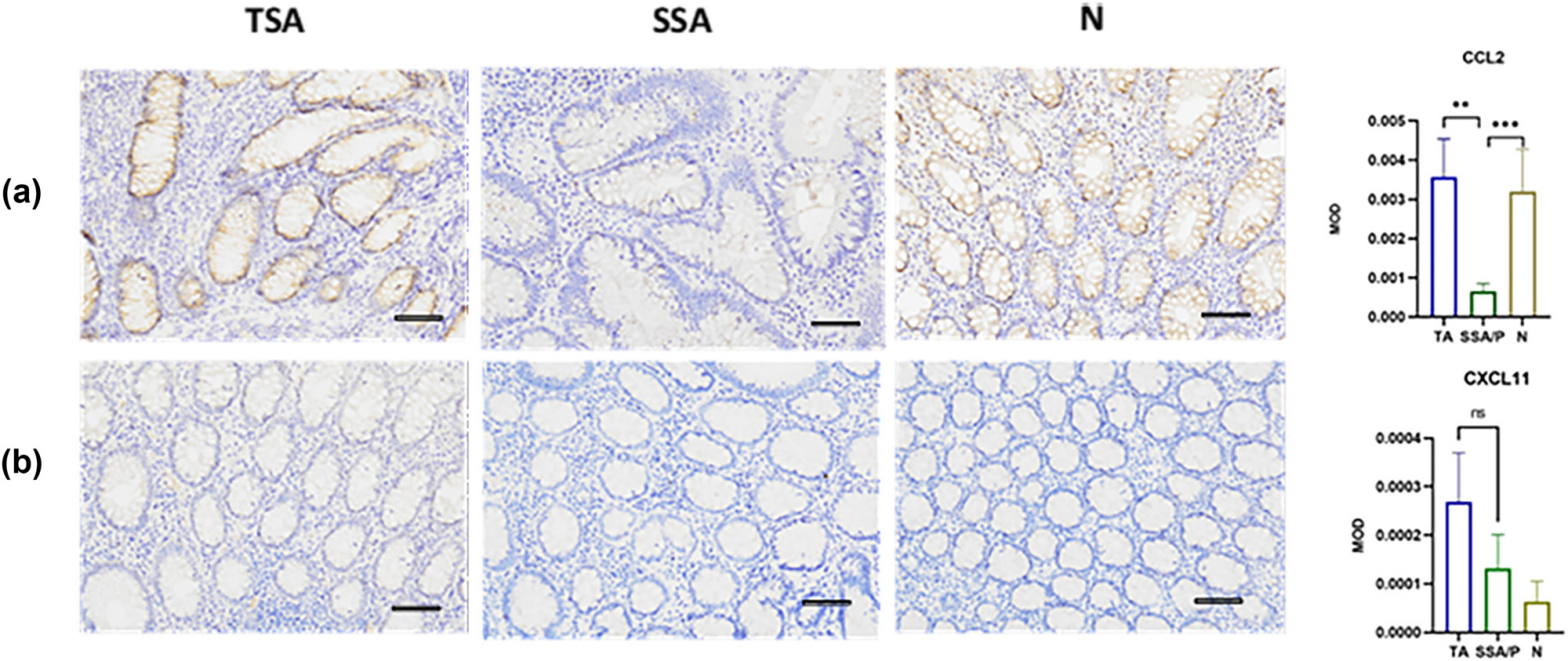
Expression of CCL2 (a) and CXCL11 (b) in TSA, normal tissues (N), and SSA. Scale bar: 200×.

We randomly selected several clinic samples for IHC analysis to further confirm the diagnostic model in clinical practice. SSA lesions displayed a lower expression of CCL2 than TSA lesions and healthy tissue (all *P* < 0.05), but there was no difference between TSA and N controls (*P* > 0.05).On the other hand, *CXCL11* was decreased in SSA and healthy tissue but drastically increased in TSA (all *P* < 0.05) (Figure 6). MCP1’s significant expression (*P* < 0.05) was consistent with the analytical data in GSE117606. According to our findings, CCL2 and CXCL11 were implicated in the pathogenesis of SSA, resulting in a new approach for SSA molecular diagnosis.

## 4 Discussion

SSA and TSA are the most common precursors of CRC; yet, distinguishing between these two subtypes may be challenging. We investigated a new molecular diagnostic model that is connected to immune molecules between SSA and TSA. Significant differences in immune cell infiltration, including activated and resting dendritic cells, activated and resting mast cells, macrophages M1, M2, and M3, regulatory T cells (Tregs), follicular helper T cells, plasma cells, activated memory T CD4 cells, and naive B cells, were observed among SSA, TSA, CRC, and healthy tissues. Similarly, a recent study [14] found enhanced cytotoxic activity of T cells and the presence of some immunosuppressive cells, including regulatory T cells, anti-inflammatory macrophages, MMP11-secreting PDGFRA + fibroblasts, and MDK + IgA + plasma cells, in early-stage serrated lesions compared to healthy colon tissue. These data suggest that early immune alterations may be important in the SSA pathway toward CRC. The discovery of two hub genes, CCL2 and CXCL11, in this study may distinguish SSA from TSA. CCL2 is a protein that regulates the recruitment of myeloid cells into inflamed sites and tumors by stimulating the chemotaxis of monocytes [15]. In certain pathological conditions, macrophages/foam cells and smooth muscle cells often exhibit increased CCL2 expression [15].

Chun et al. [16] found that CCL2 promotes CRC by enhancing the population and function of polymorphonuclear myeloid-derived suppressor cells. Feng and colleagues [17] suggested that targeting CCL2 could be an effective approach to overcome bevacizumab resistance in E26 transformation-specific variant 5 (ETV5+) CRC. Interestingly, in this study, we found increased CCL2 expression in STA lesions and healthy tissue compared to SSA lesions (all *P* < 0.05), which may imply that CCL2 is involved in the pathogenesis of SSA but not STA.

The recruitment of T cells, natural killer cells, monocytes/macrophages, and monocytes/macrophages at sites of infection is mediated by the protein-coding gene CXCL11.

This process seems to be regulated through the cognate G-protein coupled receptors CXCL1, CXCR3, and CXCL9 [18]. CXCL11 plays a role in the progression of different cancers, including head and neck cancer [19] and CRC [20,21]. CXCL11 was found to be an independent biomarker for prognosis in patients with colon adenocarcinoma [20]. In this study, we discovered that the expression of CXCL11 was decreased in SSA and healthy tissues but increased in TSA lesions, which may imply that CXCL11 is involved in the pathogenesis of TSA but not SSA, but the diagnostic value of CXCL11 requires further experimental verification.

There are a few limitations in the present study. First, CIBERSORTx is a valuable tool, but results are inferred estimates based on bulk RNA-seq and require experimental confirmation. Our result has not been verified; we will conduct verification in the subsequent experiments.

Second, the lack of basic research on SSL/SSA is the primary reason for the data shortage. Third, the software detects the immune cells; hence, a practical issue-based flow cytometric test is essential. Fourth, we verified the results by IHC; however, the clinical sample size is small, and data such as age, gender, tumor stage, and comorbidities are missing. In the subsequent research, we will add correlation analysis.

Therefore, prospective multicenter studies with large samples and functional verification test are required to assess the potential clinical application of the approach.

## 5 Conclusion

The present study provided a novel insight for diagnosing SSA and TSA, which might address the current diagnostic challenges for SSA and decline subjective judgment by the endoscopist and pathologist. CCL2 is highly promising as a potential diagnostic model for SSA and TSA; the potential diagnostic value of CXCL11 needs to be further verified.

## References

[1] Zauber AG, Winawer SJ, O’Brien MJ, Lansdorp-Vogelaar I, van Ballegooijen M, Hankey BF, et al. Colonoscopic polypectomy and long-term prevention of colorectal-cancer deaths. N Engl J Med. 2012;366(8):687–96. 10.1056/NEJMoa1100370 PMC3322371 22356322

[2] Snover DC. Update on the serrated pathway to colorectal carcinoma. Hum Pathol. 2011;42(1):1–10. 10.1016/j.humpath.2010.06.002 20869746

[3] Mezzapesa M, Losurdo G, Celiberto F, Rizzi S, d’Amati A, Piscitelli D, et al. Serrated colorectal lesions: An up-to-date review from histological pattern to molecular pathogenesis. Int J Mol Sci. 2022;23(8):4461. 10.3390/ijms23084461 PMC9032676 35457279

[4] Nosho K, Igarashi H, Ito M, Mitsuhashi K, Kurihara H, Kanno S, et al. Clinicopathological and molecular characteristics of serrated lesions in Japanese elderly patients. Digestion. 2015;91:57–63. 10.1159/000368820 25632919

[5] Longacre TA, Fenoglio-Preiser CM. Mixed hyperplastic adenomatous polyps/serrated adenomas. A distinct form of colorectal neoplasia. Am J Surg Pathol. 1990;14:524–37. 10.1097/00000478-199006000-00003 2186644

[6] Fanelli GN, Dal Pozzo CA, Depetris I, Schirripa M, Brignola S, Biason P, et al. The heterogeneous clinical and pathological landscapes of metastatic Braf-mutated colorectal cancer. Cancer Cell Int. 2020;20:30. 10.1186/s12935-020-1117-2 PMC6990491 32015690

[7] Monreal-Robles R, Jaquez-Quintana JO, Benavides-Salgado DE, Gonzalez-Gonzalez JA. Serrated polyps of the colon and rectum: a concise review. Rev Gastroenterol Mex (Engl Ed). 2021;86:276–86. 10.1016/j.rgmxen.2021.06.001 34116964

[8] Bettington M, Walker N, Clouston A, Brown I, Leggett B, Whitehall V. The serrated pathway to colorectal carcinoma: current concepts and challenges. Histopathology. 2013;62:367–86. 10.1111/his.12055 23339363

[9] Nagtegaal ID, Snover DC. Head to head: Should we adopt the term ‘sessile serrated lesion’? Histopathology. 2022;80:1019–25. 10.1111/his.14618 PMC9311759 35040174

[10] Chen B, Khodadoust MS, Liu CL, Newman AM, Alizadeh AA. Profiling tumor infiltrating immune cells with CIBERSORT. Methods Mol Biol. 2018;1711:243–59. 10.1007/978-1-4939-7493-1_12 PMC5895181 29344893

[11] Yu B, Yin YX, Tang YP, Wei KL, Pan ZG, Li KZ, et al. Diagnostic and predictive value of immune-related genes in Crohn’s Disease. Front Immunol. 2021;12:643036. 10.3389/fimmu.2021.643036 PMC8085323 33936061

[12] Yu G, Wang LG, Han Y, He QY. clusterProfiler: an R package for comparing biological themes among gene clusters. OMICS. 2012;16:284–7. 10.1089/omi.2011.0118 PMC3339379 22455463

[13] Szklarczyk D, Gable AL, Lyon D, Junge A, Wyder S, Huerta-Cepas J, et al. STRING v11: protein-protein association networks with increased coverage, supporting functional discovery in genome-wide experimental datasets. Nucleic Acids Res. 2019;47:D607–13. 10.1093/nar/gky1131 PMC6323986 30476243

[14] Zhou YJ, Lu XF, Chen H, Wang XY, Cheng W, Zhang QW, et al. Single-cell transcriptomics reveals early molecular and immune alterations underlying the serrated neoplasia pathway toward colorectal cancer. Cell Mol Gastroenterol Hepatol. 2023;15:393–424. 10.1016/j.jcmgh.2022.10.001 PMC9791140 36216310

[15] Palomino DC, Marti LC. Chemokines and immunity. Einstein (Sao Paulo). 2015;13:469–73. 10.1590/S1679-45082015RB3438 PMC4943798 26466066

[16] Chun E, Lavoie S, Michaud M, Gallini CA, Kim J, Soucy G, et al. CCL2 promotes colorectal carcinogenesis by enhancing polymorphonuclear myeloid-derived suppressor cell population and function. Cell Rep. 2015;12:244–57. 10.1016/j.celrep.2015.06.024 PMC4620029 26146082

[17] Feng H, Liu K, Shen X, Liang J, Wang C, Qiu W, et al. Targeting tumor cell-derived CCL2 as a strategy to overcome Bevacizumab resistance in ETV5+ colorectal cancer. Cell Death Dis. 2020;11(10):916. 10.1038/s41419-020-03111-7 PMC7585575 33099574

[18] Colvin RA, Campanella GS, Sun J, Luster AD. Intracellular domains of CXCR3 that mediate CXCL9, CXCL10, and CXCL11 function. J Biol Chem. 2004;279:30219–27. 10.1074/jbc.M403595200 15150261

[19] Bose S, Saha P, Chatterjee B, Srivastava AK. Chemokines driven ovarian cancer progression, metastasis and chemoresistance: Potential pharmacological targets for cancer therapy. Semin Cancer Biol. 2022;86:568–79. 10.1016/j.semcancer.2022.03.028 35378273

[20] Cao Y, Jiao N, Sun T, Ma Y, Zhang X, Chen H, et al. CXCL11 correlates with antitumor immunity and an improved prognosis in colon cancer. Front Cell Dev Biol. 2021;9:646252. 10.3389/fcell.2021.646252 PMC7991085 33777950

[21] Puchert M, Obst J, Koch C, Zieger K, Engele J. CXCL11 promotes tumor progression by the biased use of the chemokine receptors CXCR3 and CXCR7. Cytokine. 2020;125:154809. 10.1016/j.cyto.2019.154809 31437604

